# Integrated analysis of lncRNA/circRNA–miRNA–mRNA in the proliferative phase of liver regeneration in mice with liver fibrosis

**DOI:** 10.1186/s12864-023-09478-z

**Published:** 2023-07-24

**Authors:** Qian Wang, Zhangtao Long, Fengfeng Zhu, Huajian Li, Zhiqiang Xiang, Hao Liang, Yachen Wu, Xiaoming Dai, Zhu Zhu

**Affiliations:** 1 The First Affiliated Hospital, Department of Reproductive Medicine, Hengyang Medical School, University of South China, Hengyang, Hunan 421001 China; 2 The First Affiliated Hospital, Department of Hepatobiliary Surgery, Hengyang Medical School, University of South China, Hengyang, Hunan 421001 China; 3 The First Affiliated Hospital, Department of Education and Training, Hengyang Medical School, University of South China, Hengyang, Hunan 421001 China

**Keywords:** Liver fibrosis, Liver regeneration, LncRNA, CircRNA, MiRNA, MRNA

## Abstract

**Background:**

Non-coding RNAs play important roles in liver regeneration; however, their functions and mechanisms of action in the regeneration of fibrotic liver have not been elucidated. We aimed to clarify the expression patterns and regulatory functions of lncRNAs, circRNAs, miRNAs, and mRNAs in the proliferative phase of fibrotic liver regeneration.

**Methods:**

Based on a mouse model of liver fibrosis with 70% hepatectomy, whole-transcriptome profiling was performed using high-throughput sequencing on samples collected at 0, 12, 24, 48, and 72 h after hepatectomy. Hub genes were selected by weighted gene co-expression network analysis and subjected to enrichment analysis. Integrated analysis was performed to reveal the interactions of differentially expressed (DE) lncRNAs, circRNAs, miRNAs, and mRNAs, and to construct lncRNA–mRNA *cis*- and *trans*-regulatory networks and lncRNA/circRNA–miRNA–mRNA ceRNA regulatory networks. Real-Time quantitative PCR was used to validate part of the ceRNA network.

**Results:**

A total of 1,329 lncRNAs, 48 circRNAs, 167 miRNAs, and 6,458 mRNAs were differentially expressed, including 812 hub genes. Based on these DE RNAs, we examined several mechanisms of ncRNA regulatory networks, including lncRNA cis and trans interactions, circRNA parental genes, and ceRNA pathways. We constructed a *cis*-regulatory core network consisting of 64 lncRNA–mRNA pairs (53 DE lncRNAs and 58 hub genes), a *trans*-regulatory core network consisting of 103 lncRNA–mRNA pairs (18 DE lncRNAs and 85 hub genes), a lncRNA–miRNA–mRNA ceRNA core regulatory network (20 DE lncRNAs, 12 DE miRNAs, and 33 mRNAs), and a circRNA–miRNA–mRNA ceRNA core regulatory network (5 DE circRNAs, 5 DE miRNAs, and 39 mRNAs).

**Conclusions:**

These results reveal the expression patterns of lncRNAs, circRNAs, miRNAs, and mRNAs in the proliferative phase of fibrotic liver regeneration, as well as core regulatory networks of mRNAs and non-coding RNAs underlying liver regeneration. The findings provide insights into molecular mechanisms that may be useful in developing new therapeutic approaches to ameliorate diseases that are characterized by liver fibrosis, which would be beneficial for the prevention of liver failure and treatment of liver cancer.

**Supplementary Information:**

The online version contains supplementary material available at 10.1186/s12864-023-09478-z.

## Introduction

Liver fibrosis is a pathophysiological process characterized by abnormal proliferation of connective tissue in the liver, which results from chronic liver injury caused by viral hepatitis, alcoholic steatohepatitis, or non-alcoholic steatohepatitis [1]. Chronic liver injury triggers an inflammatory response, leading to the death of hepatocytes and the accumulation of extracellular matrix, resulting in the formation of scar tissue that underlies liver fibrosis [2–4]. Studies have shown that liver fibrosis can affect the reserve function and regenerative capacity of the liver [5–7]. Furthermore, liver fibrosis often progresses to liver cirrhosis, liver failure, and liver cancer, seriously affecting the prognosis and life quality of patients [8].

The liver is one of the most important organs and has a variety of complex functions such as detoxification, metabolism, biosynthesis, and immunity [9]. Additionally, the liver exhibits remarkable regenerative capabilities: in rodents with 70% hepatectomy, the residual liver rapidly restored its original size and regained its physiological functions [10, 11]. Liver regeneration is typically divided into three phases: the priming phase (0–6 h), the proliferative phase (12–72 h), and the terminal phase (96–168 h) [12]. The proliferative phase, in which quiescent (G0 phase) hepatocytes enter the cell cycle (G1/S phase) and initiate proliferation, plays a key role in liver regeneration [13]. Liver regeneration involves a variety of cells (liver parenchymal and mesenchymal cells) and molecules (cytokines, growth factors, and metabolites) and is triggered by stimuli such as surgery and injury [14–16].

Non-coding RNAs (ncRNAs) are RNA molecules that are not translated into proteins and that mainly include microRNAs (miRNAs), long non-coding RNAs (lncRNAs), and circular RNAs (circRNAs). miRNAs are single-stranded RNA molecules of 21–25 nucleotides in length that can inhibit the expression of messenger RNAs (mRNAs) by specifically binding to the 3′-untranslated region (3′-UTR) [17]. The competing endogenous RNA (ceRNA) hypothesis proposes an RNA interaction mechanism by which gene expression can be regulated by non-coding RNAs, such as lncRNAs and circRNAs, through competitively binding to miRNAs [12]. lncRNAs are RNA molecules of 200–100,000 nucleotides that regulate gene expression through *cis*-regulation, *trans*-regulation, and ceRNAs [18, 19]. Additionally, circRNAs are closed-loop RNA molecules capable of regulating gene expression through mechanisms such as regulation of parental genes, ceRNA interactions, and interactions with RNA-binding proteins [20, 21]. Studies have demonstrated that ncRNAs, including miRNAs, lncRNAs, and circRNAs, are crucial regulators of liver regeneration [22–24]. However, their functions in fibrotic liver regeneration are poorly understood.

In this study, a mouse model of liver fibrosis with 70% hepatectomy was established to identify the key mRNAs and establish lncRNA/circRNA–miRNA–mRNA regulatory networks across the proliferative phase of fibrotic liver regeneration. Whole-transcriptome profiling was performed using high-throughput sequencing. Hub genes were selected by weighted gene co-expression network analysis (WGCNA) and subjected to enrichment analysis, and differentially expressed (DE) lncRNAs, circRNAs, miRNAs, and mRNAs were subjected to correlation analysis. These findings provide new insights into the mechanisms of liver regeneration that could help identify biomarkers and therapeutic targets to ameliorate liver fibrosis.

## Materials and methods

### Establishment of a mouse model of liver fibrosis with 70% hepatectomy

Healthy adult male C57BL/6J mice (8 weeks of age and weighing 24–26 g) were purchased from Hunan SJA Laboratory Animal (Changsha, China). The mice were housed at the Experimental Animal Center of University of South China (20–25 °C, 50–55% humidity, 12 h light/12 h dark, standard chow, and free access to water and food). Carbon tetrachloride in olive oil was injected intraperitoneally at 5 ml/kg twice a week for 8 weeks. Thirty mice underwent 70% hepatectomy at week 9 by excision of the left lateral and middle lobes after intraperitoneal injection of l% pentobarbital sodium at 50 mg/kg [25]. The liver samples were fixed in 4% paraformaldehyde for histopathological examination using hematoxylin and eosin and Masson staining. To assess hepatocyte proliferation, the sections were further processed for immunohistochemistry with anti-Ki-67 antibody (1:100, Abcam, United Kingdom). The right lateral lobes were collected at 0, 12, 24, 48, and 72 h after hepatectomy with six mice in each group. The samples were stored at − 80 °C within 30 min following collection. All animal experiments were performed in accordance with internationally recognized guidelines for the care and use of laboratory animals and were approved by the Committee for the Care and Use of Experimental Animals of University of South China.

### High-throughput RNA sequencing

Liver samples of mice with liver fibrosis were sent to Novogene (Beijing, China) for high-throughput sequencing. Strand-specific libraries were generated using the NEBNext® UltraTM RNA Library Prep Kit for Illumina® (NEB, USA). Total RNA was extracted, and the purity, integrity and concentration were evaluated by 1% agarose gel electrophoresis and *OD*_260/280_ and *OD*_260/230_ measurement (NanoPhotometer® spectrophotometer; Implen, Germany). The Agilent 2100 bioanalyzer (Agilent Technologies, USA) was used to further verify the RNA integrity and quality. Total RNA (1 µg) of each sample was used for transcriptome sequencing, and double-stranded cDNA was synthesized using fragmented rRNA-free RNA as a template. The purified double-stranded cDNA was subjected to end repair, poly-A addition, and adaptor ligation. Resulting cDNA fragments of 350–400 bp were selected using AMPure XP beads (Beckman Coulter, USA), and the second cDNA strand was degraded. Finally, PCR amplification was performed to construct the library, and the effective concentration (> 2 nM) was measured by real-time quantitative PCR (RT-qPCR) to ensure the quality.

The construction of miRNA libraries followed a similar, but slightly different procedure. The small RNA libraries were generated using the NEBNext® Multiplex Small RNA Library Prep Set for Illumina® (NEB, USA). Briefly, 2 µg of total RNA from each sample was used to prepare the miRNA libraries. The purified miRNA was ligated with adaptors at the 3ˊ and 5ˊ ends, and the first cDNA strand was synthesized using reverse transcription primers. After PCR amplification of the cDNA library, the products were purified, and 140–160 bp DNA fragments were recovered. Finally, the quality of the library was assessed using an Agilent 2100 bioanalyzer (effective library concentration > 2 nM).

Quality-verified libraries were used for sequencing with PE150 on an Illumina NovaSeq 6000 platform (Illumina, USA) based on the effective concentration of the library and the requirements of data output. Raw reads were filtered using Perl 6 to remove reads with adaptors, undetermined bases at a frequency of > 0.002, or > 50% low-quality bases at one end, in order to ensure the quality and reliability of the sequencing data. The error rates (Q20 and Q30) and GC content were determined using Illumina Casava (v1.8) to obtain clean reads for subsequent analysis (Q20 > 95%, Q30 > 90%, GC = 48–52%).

The reference genome (GRCm39) and gene model annotation files of lncRNAs and mRNAs were downloaded from the genome website (https://www.ncbi.nlm.nih.gov/). Clean reads were aligned against the reference genome using hisat (v2.0.5), and the mapped read count was calculated using StringTie (v1.3.3). circRNAs were identified by find_circ (v1.0) [26] and CIRI (v2.0.5) [27]. Clean reads were mapped to the reference sequences in miRBase (v22.0) using Bowtie (v2.0.6) to identify known miRNA sequences. The sQuantifier.pl script in MirDeep2 was used to quantify miRNAs and obtain read counts.

### Identification of differentially expressed (DE) RNA genes

To quantify the gene expression levels, the read counts were normalized using the transcripts per million method. Violin plots were used to visualize the overall distributions of gene expression levels. The heatmap R package (v1.0.12) was used to generate heatmaps.

The lncRNAs, circRNAs, miRNAs, and mRNAs with differential expression in the 12 h, 24, 48 and 72 h groups relative to the 0 h group were selected using the edge package (v3.38.4) in R (v4.1.0) [28]. Read counts were normalized using trimmed mean of M-values, and DE genes were selected based on the negative binomial distribution test. For lncRNAs, miRNAs, and mRNAs, the selection criteria were |log2 (Fold Change)| > log2 (1.5) and *p* < 0.05 for DE genes; log2 (Fold Change) > log2 (1.5) for significantly up-regulated genes; and log2 (Fold Change) < − log2 (1.5) for significantly down-regulated genes. For circRNAs, the selection criteria were |log2 (Fold Change)| > log2 (1) and *p* < 0.05 for DE genes; log2 (Fold Change) > log2 (1) for significantly up-regulated genes; and log2 (Fold Change) < − log2 (1) for significantly down-regulated genes. Finally, the ggplot2 R package (v3.0.4) was used to generate volcano plots.

### Weighted gene co-expression network analysis

A weighted gene co-expression network was created using the WGCNA R package (v1.71). The read count of DE mRNA was normalized using the fragments per kilobase million method for clustering. A soft threshold was determined after removing outliers. An adjacency matrix was constructed and transformed into a topological overlap matrix. DE mRNAs were clustered and divided into modules using the dynamic tree cutting algorithm. Module–phase correlations were calculated, and the modules with the highest correlations were selected as key modules.

### Identification and functional enrichment of hub genes

The gene significance (GS, indicative of the correlation between the gene expression pattern and the trait/regeneration phase), and the module membership (MM, indicative of the correlation between the gene expression pattern and module eigengene) were calculated. The correlations between GS and MM were analyzed for key modules and presented in scatter plots. The mRNAs with |MM| >0.8 and |GS| >0.5 in key modules were selected as hub genes for further analysis.

The R package ClusterProfilter (v4.4.4) was used for Gene Ontology [29] and Kyoto Encyclopedia of Genes and Genomes [30–33] enrichment analysis of the hub genes. GO enrichment analysis included biological processes, cellular components, and molecular functions, but only biological processes are shown in the present study. Gene annotation was performed using the org.Mm.eg.db R package (v3.15.0). The *p*-values in GO/KEGG enrichment analysis were calculated based on a hypergeometric distribution and corrected using the false discovery rate (FDR), and *p* < 0.05 indicated significant enrichment. Finally, the ggplot2 R package (v3.0.4) was used to visualize the top ten terms/pathways based on the *p*-value.

### lncRNA/circRNA–mRNA regulatory networks and functional enrichment

Pearson correlation coefficients (*r*) between DE lncRNAs or DE circRNAs and their potential regulatory targets were calculated using the Hmisc R package (v4.7-1). DE mRNAs located within 100 kb upstream and downstream of DE lncRNAs on the same chromosome were selected as potential *cis*-regulatory targets. The lncRNA–mRNA and circRNA-mRNA pairs with |*r*| >0.6 were selected as *cis*-regulatory pairs, and lncRNA–mRNA pairs with |*r*| >0.9 were selected as *trans*-regulatory pairs. The mRNAs were subjected to GO/KEGG enrichment analysis, and the top ten terms/pathways for the *cis*-regulatory and *trans*-regulatory pairs were visualized based on the *p*-values. The regulatory pairs containing hub genes were selected as key pairs, and corresponding lncRNA/circRNA–mRNA *cis*-regulatory and *trans*-regulatory core networks were constructed.

### Construction of ceRNA core networks

Starbase [34] and miRanda (v3.3a) were used to predict the potential targets of DE lncRNA–DE miRNA and hub gene–DE miRNA pairs; miRanda (v3.3a) was used to predict the potential targets of DE circRNA–DE miRNA pairs. lncRNA/circRNA–miRNA–mRNA (hub gene) ceRNA regulatory networks were constructed according to the following criteria: (1) one or more DE miRNA binding sites shared by DE lncRNAs/DE circRNAs and hub genes; (2) positive correlations between the expression of DE lncRNAs/DE circRNAs and hub genes sharing the DE miRNA binding sites; and (3) negative correlations between the expression of DE miRNAs, target DE lncRNAs and hub genes. lncRNA–miRNA–mRNA and circRNA–miRNA–mRNA ceRNA core regulatory networks were constructed based on the Spearman correlation coefficient (*ρ* < 0.05).

### Real-time quantitative PCR

One lncRNA–miRNA–mRNA pathway and one circRNA–miRNA–mRNA pathway were randomly selected from the ceRNA regulatory networks, and the mRNAs in both pathways were detected by RT-qPCR for validation. RNA was extracted using the TRIzol method, and cDNA was reverse transcribed using total mRNA as a template. PCR was performed using SYBR Green PCR Master Mix and specific primers (Supplementary Table S1). Relative gene expression was calculated using the 2^−ΔΔCt^ method. The read counts from high-throughput RNA sequencing were normalized using the transcripts per million method to obtain the relative expression of RNA for comparison with the RT-qPCR results. One-way analysis of variance was used for comparisons between groups. The Bonferroni method was used to adjust the p-value, and the significant level set at *P* < 0.05. The expression patterns of ncRNAs and mRNAs in the two pathways were assessed to determine whether they conformed to the ceRNA hypothesis.

## Results

### Analysis of lncRNAs, circRNAs, miRNAs, and mRNAs that are differentially expressed during liver regeneration

**Fig. 1:**
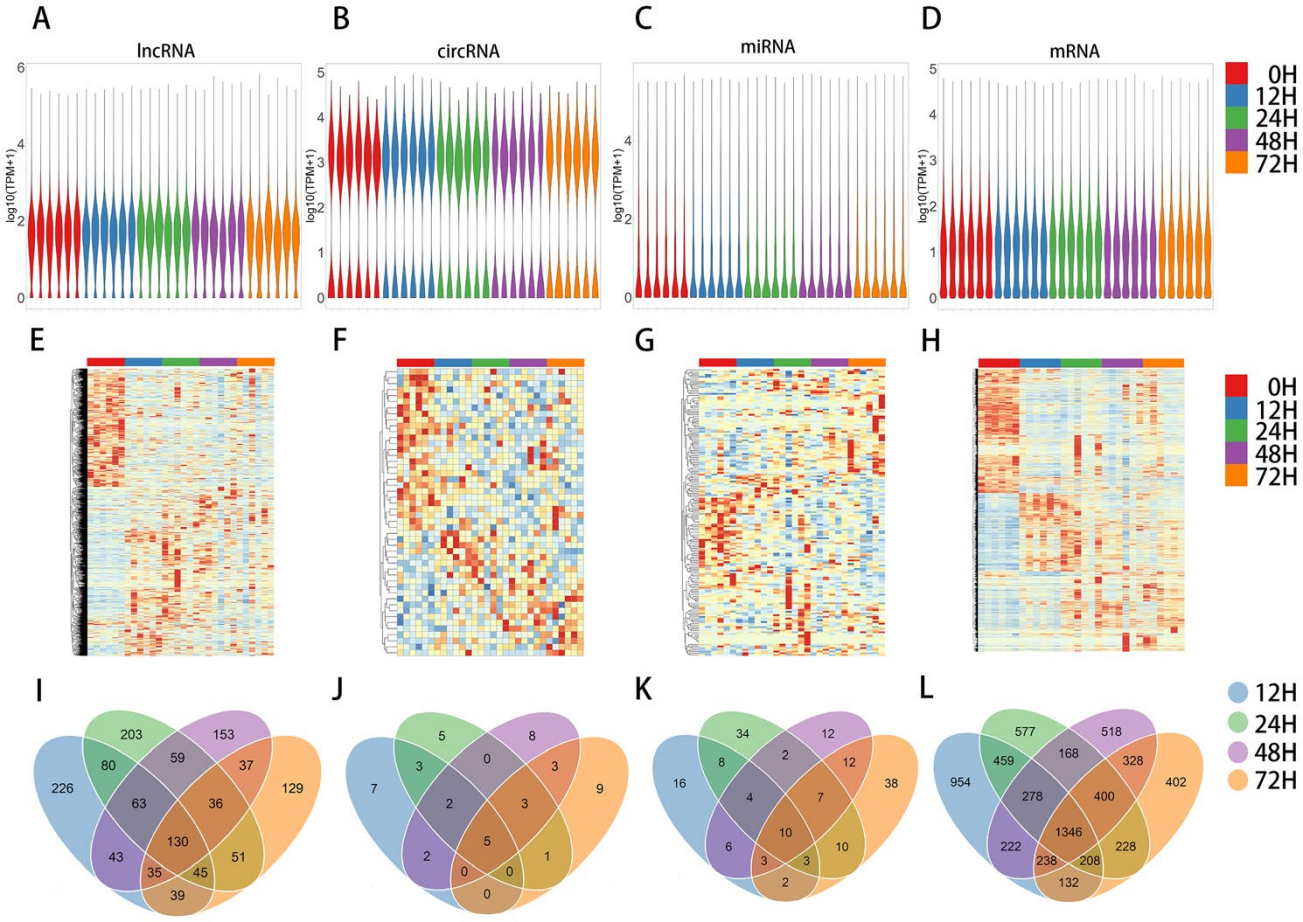
Expression patterns of lncRNAs, circRNAs, miRNAs, and mRNAs during liver regeneration in mice with liver fibrosis. **A-D** Violin plots of lncRNA expression (**A**), circRNA expression (**B**), miRNA expression (**C**), and mRNA expression (**D**). **E-H** Hierarchical clustering heatmap of lncRNAs (**E**), circRNAs (**F**), miRNAs (**G**), and mRNAs (**H**). **I-L** Venn diagram of lncRNAs (**I**), circRNAs (**J**), miRNAs (**K**), and mRNAs (**L**). Panels A-H show the expression profiles in 0 h, 12 h, 24 h, 48 h, and 72 h groups of mice; and panels I-L show differentially expressed (DE) RNAs in the proliferation phase (12–72 h) groups relative to the 0 h group

To identify regulatory networks that underlie the proliferative phase of fibrotic liver regeneration, we established a mouse model of liver fibrosis with 70% hepatectomy. Subsequent examination revealed that all mice exhibited the pathological features of liver fibrosis (Supplementary Fig. 1). Total RNA was extracted from liver samples at 0, 12, 24, 48, and 72 h for high-throughput sequencing and quantitative analysis. After excluding genes with no expression in 25% of samples, 3,706 lncRNAs, 185 circRNAs, 563 miRNAs, and 14,999 mRNAs were identified (Supplementary Table S2), for which the expression patterns are presented as violin plots (Fig. 1A–D) and heatmaps (Fig. 1E–H).

Compared with the 0 h group, the proliferation phase groups (12–72 h) showed the following differentially expressed genes: 661 lncRNAs (352 up-regulated and 309 down-regulated), 19 circRNAs (9 and 10), 52 miRNAs (35 and 17), and 3,837 mRNAs (1,795 and 2,042) in the 12 h group; 667 lncRNAs (358 and 309), 19 circRNAs (7 and 12), 78 miRNAs (65 and 13), and 3,664 mRNAs (2,107 and 1,557) in the 24 h group; 556 lncRNAs (283 and 273), 23 circRNAs (10 and 13), 56 miRNAs (39 and 17), and 3,498 mRNAs (1,978 and 1,520) in the 48 h group; and 502 lncRNAs (274 and 228), 21 circRNAs (9 and 12), 85 miRNAs (61 and 24), and 3,282 mRNAs (1,982 and 1,300) in the 72 h group (Supplementary Table S3 and Supplementary Fig. 2). The RNAs with differential expression at any time point after 70% hepatectomy were considered to be DE genes. Together, these included 1,329 lncRNAs, 48 circRNAs, 167 miRNAs, and 6,458 mRNAs (Fig. 1I–L), representing significant changes in the proliferative phase of liver regeneration in mice with liver fibrosis.

### Identification of key modules and hub genes associated with liver regeneration

**Fig. 2:**
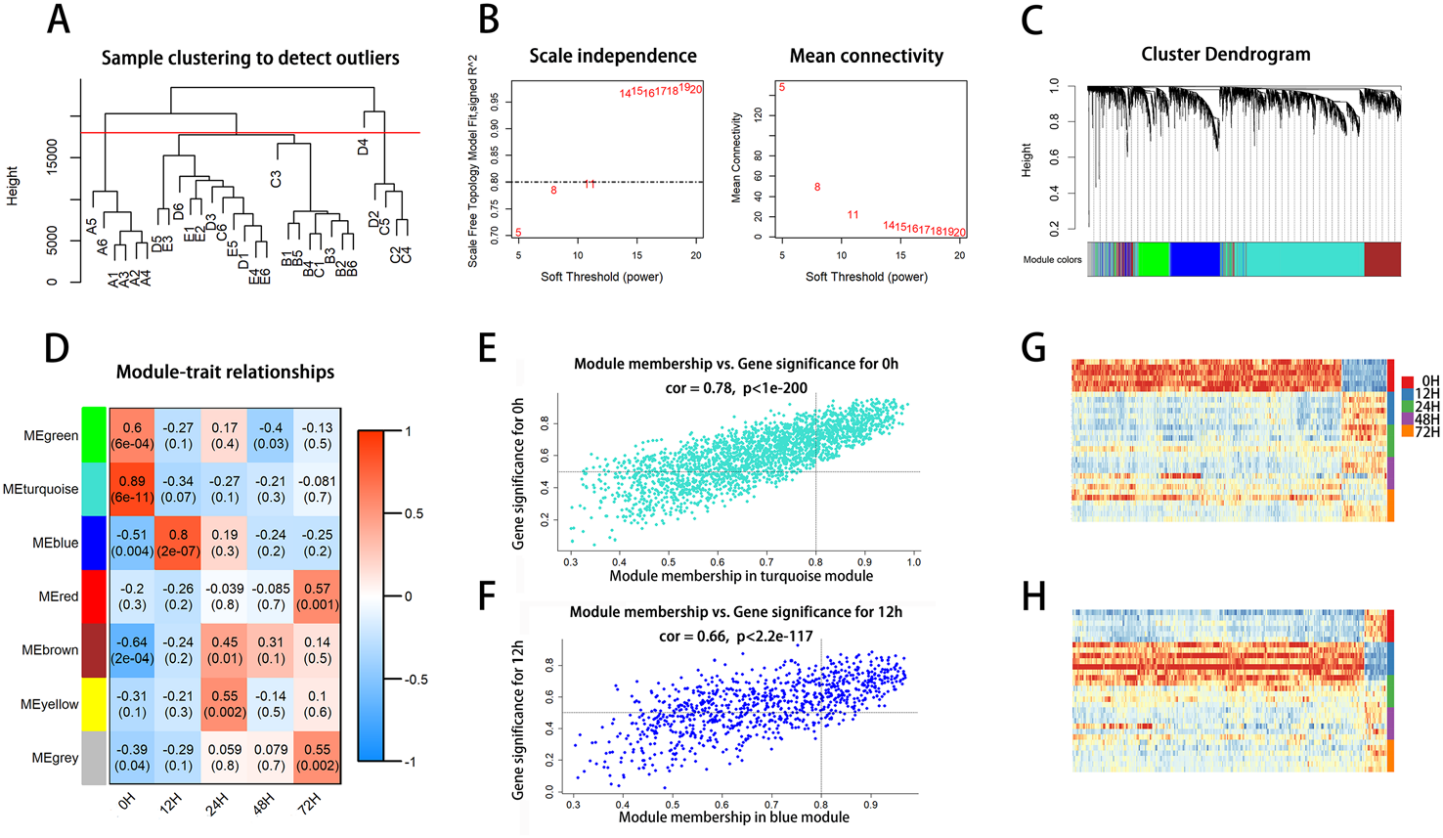
Weighted gene co-expression network analysis of differentially expressed genes during liver regeneration. **A** Clustering of genes that were diffentially expressed in the 12 h, 24 h, 48 h, and 72 h groups as compared to the 0 h group. **B** Soft threshold power for topological analysis. **C** Cluster dendrogram. **D** Hierarchical clustering heatmap. **E-F** Scatter plot of correlations between module membership (MM) and gene significance (GS) in MEturquoise (**E**) and MEblue (**F**). **G-H** Clustering heatmaps of DE hub genes in MEturquoise (**G**) and MEblue (**H**)

To further evaluate patterns of expression of the DE RNAs during liver regeneration, we performed weighted gene co-expression network analysis (WGCNA). After normalizing the read counts of 6,458 DE mRNAs, the outlier sample D4 was removed (Fig. 2A), and the soft threshold was set to 11 (Fig. 2B). The remaining DE mRNAs were then divided into 7 modules based on their expression patterns. These included MEturquoise (2,289 DE mRNAs), MEblue (930), MEgreen (548), MEgray (542), MEred (448), MEyellow (788), and MEbrown (832) (Fig. 2C and Supplementary Table S4). The correlations between co-expression modules and each regeneration phase were determined (Fig. 2D), and the MEturquoise (0 h) and the MEblue (12 h) with the highest correlations were selected as key modules.

Next, we sought to select and functionally enrich hub genes within the key modules. Among 3,219 DE mRNAs within the two key modules, scatter plots showed significant correlations between gene significance (GS) and module membership (MM) (Fig. 2E–F). A total of 812 hub genes were selected. This included 556 hub genes in MEturquoise, of which 79 were significantly up-regulated and 477 were significantly down-regulated at 0 h (Fig. 2G). Additionally, 256 hub genes in MEblue, of which 238 were significantly up-regulated and 18 were significantly down-regulated at 12 h (Fig. 2H).

### Functional enrichment of hub genes associated with liver regeneration

**Fig. 3:**
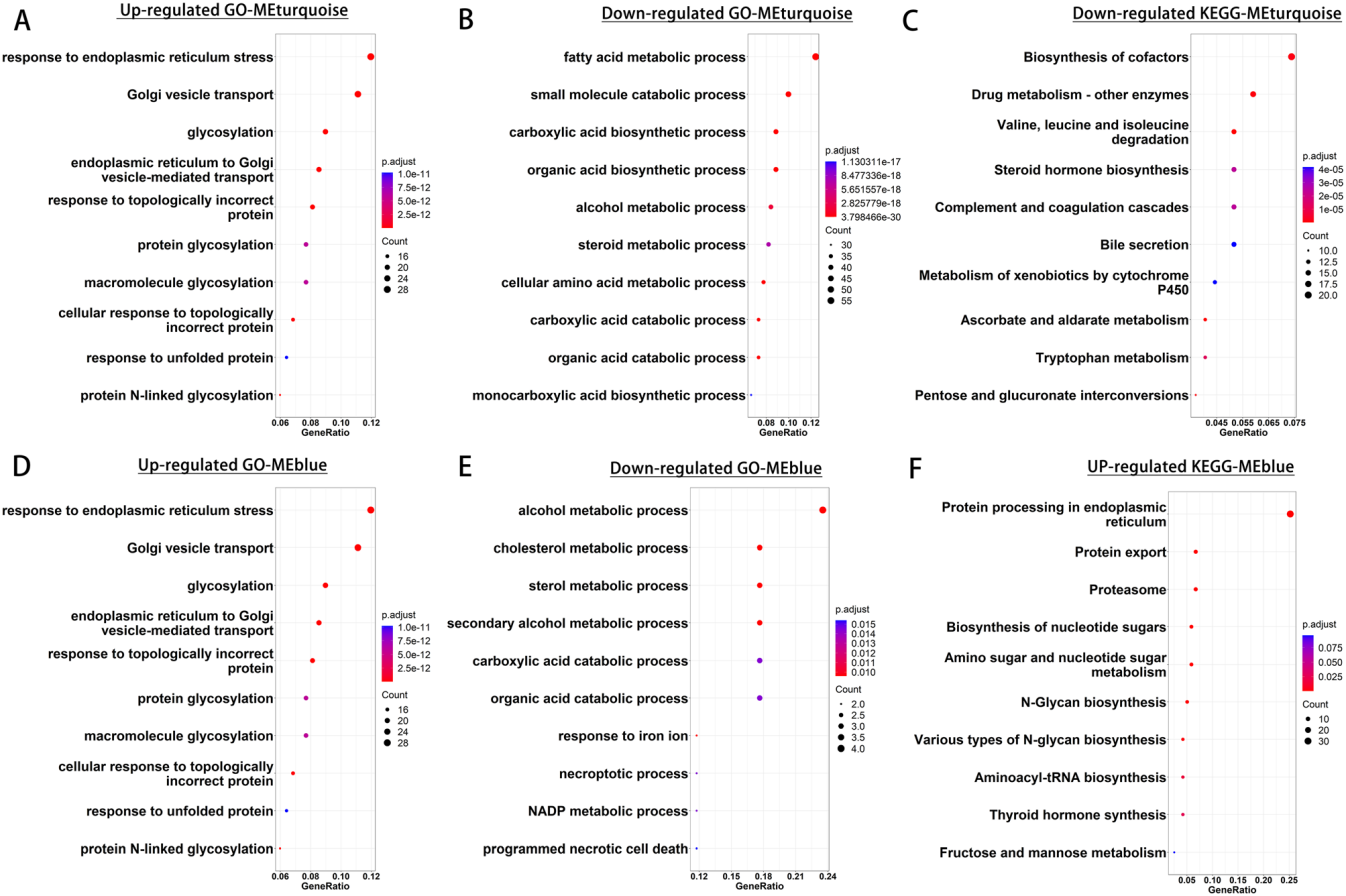
Pathway analysis of hub genes. **A–C** GO/KEGG enrichment analysis of hub genes in MEturquoise. **A** GO enrichment analysis of up-regulated hub genes. **B** GO enrichment analysis of down-regulated hub genes. **C** KEGG enrichment analysis of down-regulated hub genes. **D–F** GO/KEGG enrichment analysis of hub genes in MEblue. **D** GO enrichment analysis of up-regulated hub genes. **E** GO enrichment analysis of down-regulated hub genes. **F** KEGG enrichment analysis of up-regulated hub genes

To determine the function of the hub genes in ME turquoise and MEblue, we performed GO and KEGG enrichment analyses. For GO analysis, the up-regulated hub genes in MEturquoise were mainly associated with regulation of cell growth, extracellular matrix organization, and extracellular structure organization (Fig. 3A); and the down-regulated hub genes were primarily associated with fatty acid metabolic process, small molecule catabolic process, and cellular amino acid metabolic process (Fig. 3B). For KEGG analysis, the up-regulated hub genes in MEturquoise showed no significant KEGG enrichment, and the down-regulated hub genes were enriched in valine, leucine and isoleucine degradation, steroid hormone biosynthesis, and pentose and glucuronide interconversions (Fig. 3C).

For MEblue, GO enrichment analysis indicate that the up-regulated hub genes were mainly associated with response to endoplasmic reticulum stress, Golgi vesicle transport, and endoplasmic reticulum to Golgi vesicle-mediated transport (Fig. 3D). The down-regulated hub genes were primarily associated with cholesterol metabolic process, sterol metabolic process, and secondary alcohol metabolic process (Fig. 3E). In KEGG enrichment analysis, the up-regulated hub genes in MEblue were enriched in protein export, amino sugar and nucleotide sugar metabolism, and fructose and mannose metabolism (Fig. 3F), and the down-regulated hub genes showed no significant KEGG enrichment. These pathways are consistent with increased metabolic proliferative activity and extracellular matrix remodeling, both of which are associated with liver regeneration.

### Identification and functional enrichment of core lncRNA/circRNA–mRNA regulatory pairs associated with liver regeneration

**Fig. 4:**
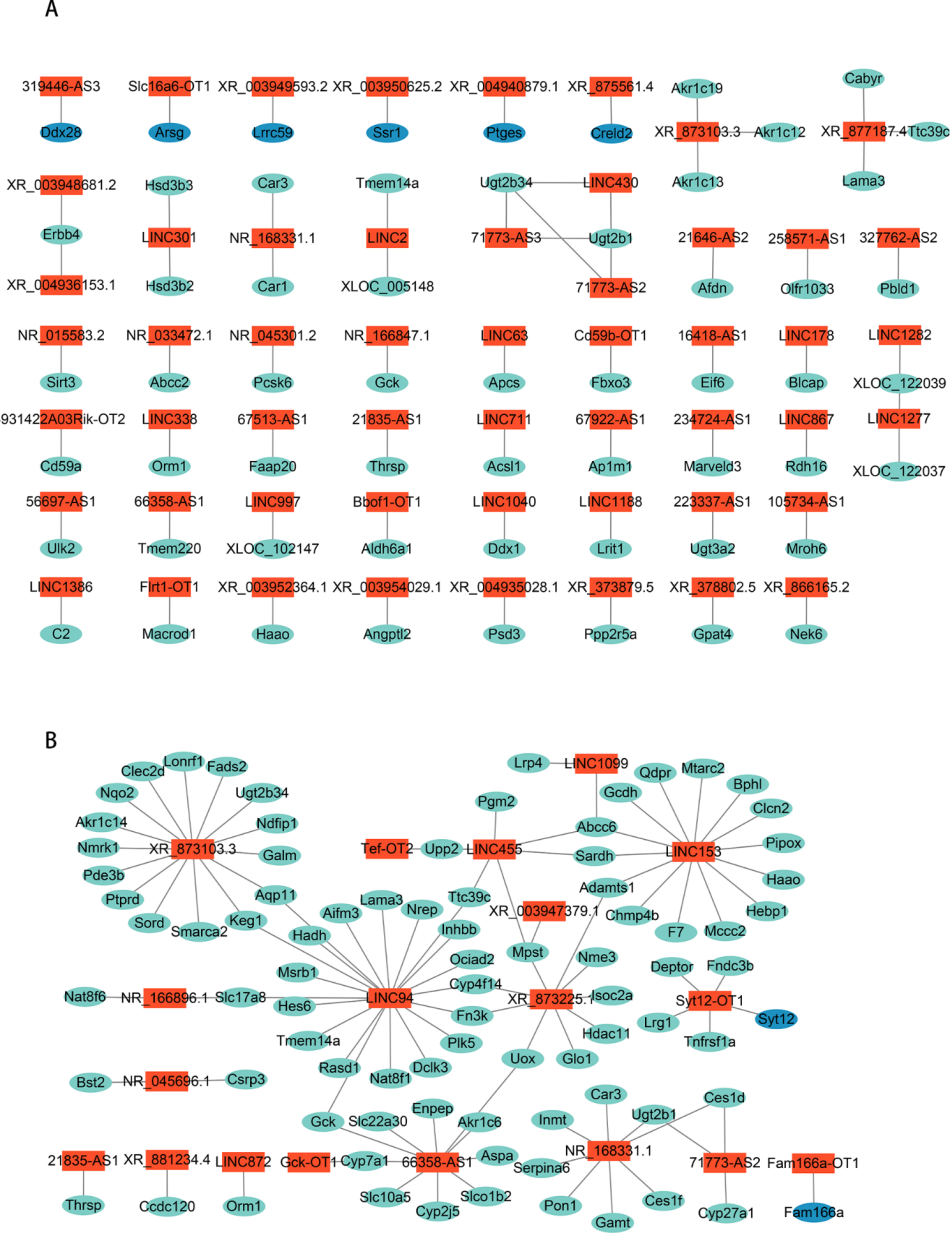
Identification of lncRNA-mRNA and circRNA-mRNA core pairs. **A** lncRNA–mRNA *cis*-regulatory core network. **B** lncRNA–mRNA *trans*-regulatory core network. Red, lncRNA; turquoise, hub genes in MEturquoise; and blue, hub genes in MEblue.

Given the key regulatory roles of lncRNAs and circRNAs in ceRNA core networks, we evaluated the relationship between lncRNAs/cirRNAs and mRNAs in terms of *cis*- and *trans*-regulation potential. For lncRNAs, a total of 175 DE lncRNAs and 190 DE mRNAs were identified, which formulated 212 potential *cis*-regulatory pairs and 103 core *trans*-regulatory pairs. Based on hub genes and their corresponding lncRNAs, 64 core *cis*-regulatory pairs consisting of 53 DE lncRNAs and 58 hub genes were identified (Fig. 4A). GO analysis revealed enrichment in steroid metabolic process, ribonucleotide metabolic process, and purine ribonucleotide metabolic process (Supplementary Fig. 3A); KEGG analysis showed enrichment in steroid hormone biosynthesis, bile secretion, and ascorbate and aldarate metabolism (Supplementary Fig. 3B). The 103 core *trans*-regulatory pairs consisted of 18 DE lncRNAs and 85 hub genes (Fig. 4B). GO analysis showed enrichment in small molecule catabolic process, organic acid catabolic process, and steroid metabolic process (Supplementary Fig. 3C). KEGG analysis showed enrichment in valine, leucine and isoleucine degradation, fatty acid degradation, and tryptophan metabolism (Supplementary Fig. 3D).

For circRNAs, a total of 48 potential circRNA–parent gene pairs were identified. GO analysis of parent genes showed enrichment in small molecule catabolic process, deoxyribonucleotide metabolic process, and nucleoside metabolic process (Supplementary Fig. 3E), while KEGG analysis showed no significantly enriched pathways.

### Construction of ceRNA core networks

**Fig. 5:**
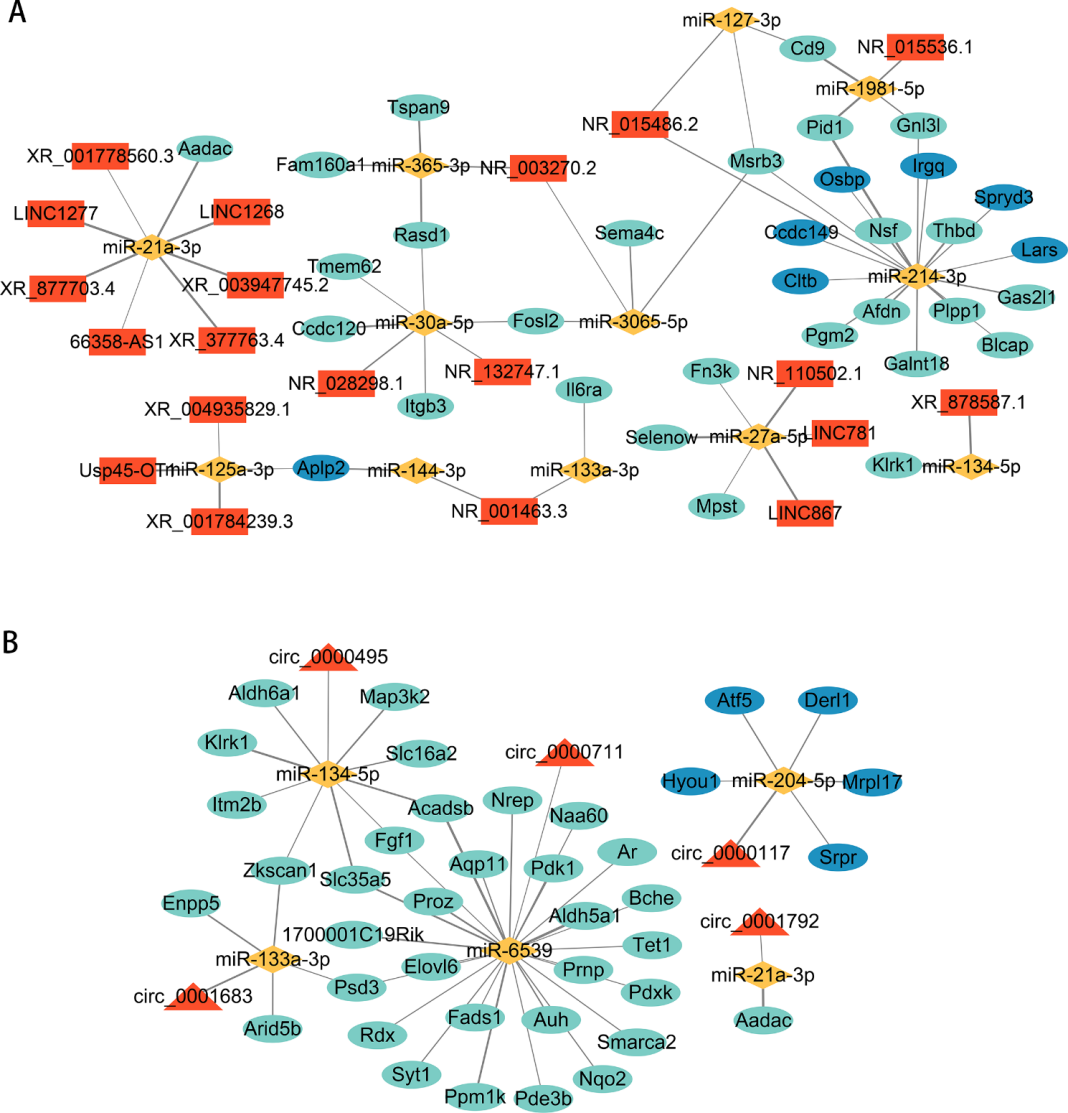
Identification of ceRNA core regulatory networks. (**A**) lncRNA–miRNA–mRNA core network. (**B**) circRNA–miRNA–mRNA core network. Red, lncRNA/circRNA; yellow, miRNA; turquoise, hub genes in MEturquoise; blue, hub genes in MEblue.

Next, we evaluated functional interactions based on the set of 167 DE miRNAs that were differentially expressed in the 12 h, 24 h, 48 h, or 72 h group as compared to the 0 h group. The Starbase database was used to predict the targets of the DE miRNAs, which included 199,946 DE miRNA–DE mRNA pairs and 1,456 DE miRNA–lncRNA pairs (Supplementary Table S5). Additionally, miRanda software was used to identify 29,237 DE miRNA–DE mRNA pairs; 643,159 DE miRNA–lncRNA pairs; and 76,407 DE circRNA–DE miRNA pairs (Supplementary Table S6). Based on the identified hub genes and the ceRNA hypothesis, we constructed a lncRNA–miRNA–mRNA ceRNA core regulatory network (Fig. 5A), which contained 20 lncRNAs, 12 miRNAs, and 33 hub genes. We also constructed a circRNA–miRNA–mRNA ceRNA core regulatory network (Fig. 5B), which contained 5 circRNAs, 5 miRNAs, and 39 hub genes.

**Fig. 6:**
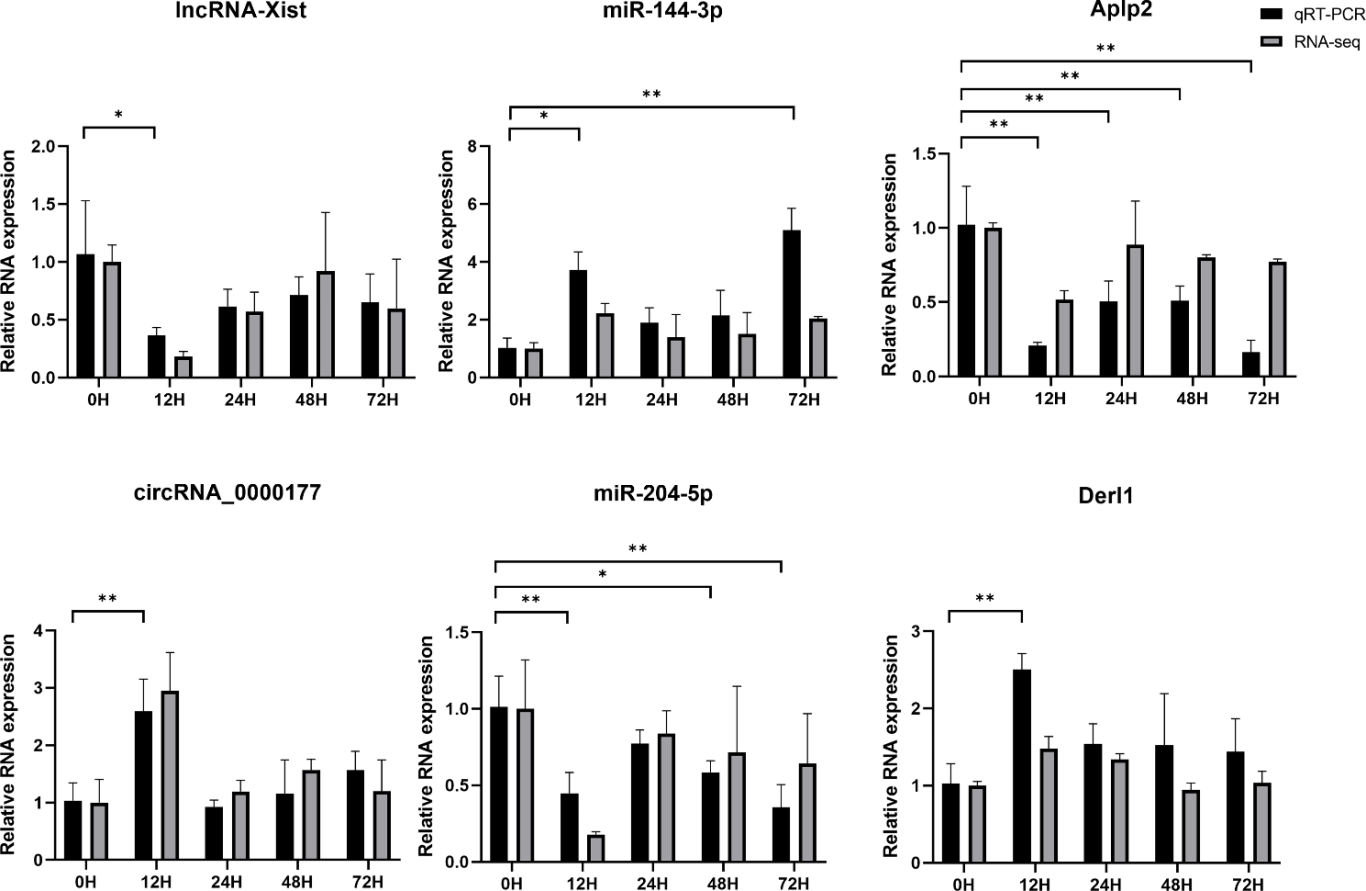
Releative expression levels of lncRNAs, circRNAs, miRNAs, and mRNAs in ceRNA networks. The black bar shows the qRT-PCR data and the the gray bar shows the RNA-seq data. *P < 0.05 and **P < 0.01 for comparisons between groups

As verification of the ceRNA core networks, we randomly selected RNAs in two pathways that are regulated in response to liver regeneration for RT-qPCR analysis. This includes *lncRNA-Xist* (*NR_001463.3*)/*miR-144-3P*/*mRNA-Aplp2*, which was identified to be downregulated, and *circRNA-0000117*/*miRNA-204-5p*/*mRNA-Derl1*, which was identified to be upregulated in response to liver regeneration. Their relative expression levels measured by RT-qPCR corresponded with the results of high-throughput RNA sequencing, and the change in the patterns of ncRNAs and mRNAs in both pathways, which was most obvious at 12 h but was also showed a similar trend at other time points, conformed to the ceRNA hypothesis (Fig. 6).

## Discussion

Current transcriptome-wide studies on liver regeneration have focused on healthy livers [25, 35]. This study presents the first analysis of expression patterns and mechanisms of action of lncRNAs, circRNAs, miRNAs, and mRNAs in the regeneration of fibrotic liver, which may provide new insights for the development of therapeutic strategies for liver diseases. Liver fibrosis is a common pathological process that occurs in a variety of chronic liver diseases [36], and the injury stimuli and molecular signals are likely to be different for liver fibrosis and regeneration. Therefore, we reasoned that the transcriptome-wide study of liver regeneration in the presence of fibrosis may help to elucidate the molecular mechanisms underlying curative processes in the disease and provide new targets for preclinical investigations.

In this study, we employed WGCNA, a systems biology approach for describing the correlation patterns among genes [37], to effectively screen for hub genes closely associated with phenotypes. MEturquoise (0 h) and MEblue (12 h) had the highest correlations with fibrotic liver regeneration, suggesting that the expression of hub genes exhibited the most significant changes in the early stage of liver regeneration. Using an ischemia-reperfusion 85% hepatectomy mouse model and mRNA microarray analysis, Liu et al. [38] performed WGCNA and showed that the changes of hub gene expression were most significant during 6–24 h after hepatectomy. Furthermore, Zhu [39] conducted WGCNA based on a rat portal vein ligation model and transcriptome-wide sequencing and revealed that the changes in hub gene expression were most significant at 0 h after operation. These findings are consistent with and further support our results.

We also performed a series of functional enrichment analyses based on bioinformatic knowledge from GO and KEGG pathway databases. The results demonstrated that the up-regulated hub genes were mainly associated with biological processes, such as regulation of cell growth, protein and nucleotide processing and synthesis, and extracellular matrix organization, which is consistent with liver regeneration being primarily driven by hepatocyte proliferation [40]. Cellular proliferation requires a significant amount of nucleotides for the synthesis of RNAs involved in transcription and translation [41], and regulatory proteins such as cytokines and growth factors also play crucial roles [42]. Moreover, the extracellular matrix provides a framework for hepatocytes and maintains homeostasis in the liver [43]. Over-deposition of diffuse extracellular matrix is a key feature of liver fibrosis, and extracellular matrix degradation and remodeling are important steps in liver regeneration [44]. The down-regulated hub genes in this study were mainly enriched in biological processes such as fatty acid metabolic process, cholesterol metabolic process, steroid hormone biosynthesis, and branched-chain amino acid degradation. Lipid metabolism has an important role in liver regeneration [42, 45]. Indeed, fatty acid oxidation is the main source of energy for liver regeneration, and inhibition of fatty acid β-oxidation has been reported to delay liver regeneration after hepatectomy [46]. Cholesterol affects liver regeneration by regulating cell cycle progression [47], and steroids inhibit liver regeneration by suppressing TNF-α and IL-6 overproduction and hepatocyte DNA synthesis [48, 49]. Moreover, amino acids also have a crucial role in liver regeneration. Valine, leucine, and isoleucine, which are collectively known as branched-chain amino acids, can promote liver regeneration by enhancing hepatocyte growth factor secretion and protein synthesis [50, 51]. Therefore, our findings are consistent with processes that are known to underlie liver regeneration.

In the present study, lncRNA/circRNA–miRNA–mRNA regulatory networks were constructed by investigating the mechanisms of lncRNA *cis* and *trans* interactions, circRNA parental genes, and ceRNA interactions. Compared to existing transcriptome-wide studies on liver regeneration, this work provides a more comprehensive analysis of the mechanisms of action of ncRNAs [52, 53]. Among the lncRNA/circRNA–miRNA–mRNA ceRNA core regulatory networks identified in our study, we selected two pathways for RT-qPCR validation: *lncRNA-Xist*/*miR-144-3P*/*mRNA-Aplp2*, which was downregulated by liver regeneration; and *circRNA-0000117*/*miRNA-204-5p*/*mRNA-Derl1*, which was upregulated. Our results show that the relative expression levels of the RNAs in these pathways were highly consistent with the results of high-throughput RNA sequencing. Notably, the change patterns of ncRNAs and mRNAs in the two pathways conformed to the ceRNA hypothesis, which further affirms the reliability of our analysis. Studies have shown that *lncRNA-XIST* promotes the proliferation of pancreatic and lung cancer cells by targeting and inhibiting *miR-133a* and *miR-144-3p*, respectively [54, 55]. Moreover, *circ-0000117* promotes the proliferation of gastric cancer cells by inhibiting the *miR-337-3p*/*STAT3* axis [56]. Studies have also demonstrated that *miR-204* down-regulates the expression of *Bcl-2*, *Sirt1*, and *Fn-1* to inhibit the proliferation and promote the apoptosis of hepatoma cells and tumor endothelial cells [57, 58]. Taken together, we speculate that the above pathways also have important roles in liver regeneration by regulating cell proliferation and apoptosis.

There are some limitations in our study. First, liver fibrosis can arise from a range of chronic liver diseases, such as viral hepatitis, alcoholic hepatitis, non-alcoholic fatty liver, and autoimmune hepatitis. While our model demonstrated consistent liver fibrosis features, it is incapable of simulating all pathological alterations resulting from different diseases. Second, although we examined several mechanisms of ncRNA regulatory networks, such as lncRNA *cis* and *trans* interactions, circRNA parental genes, and ceRNA pathways, there is still a need for further exploration of additional mechanisms, including protein binding and interaction, due to the intricate nature of ncRNA mechanisms. Finally, through bioinformatics analysis, we predicted the regulatory networks of ncRNAs and hub genes during the proliferative phase of liver regeneration. However, the functions of the core networks and pathways across the time spectrum need to be experimentally verified in the future.

## Conclusions

In this study, we revealed the expression patterns of lncRNAs, circRNAs, miRNAs, and mRNAs in the proliferative phase of liver regeneration in mice with liver fibrosis. We identified hub mRNAs and constructed lncRNA/circRNA–miRNA–mRNA regulatory networks. This study contributes to the understanding of the molecular mechanisms of fibrotic liver regeneration. Our findings provide new insights into the process of liver regeneration and potential targets for preclinical studies.

## Electronic supplementary material

Below is the link to the electronic supplementary material.

**Additional file 1: Supplementary Table S1**. Sequences of PCR primers.

**Additional file 2: Supplementary Table S2**. Summary of all lncRNAs, circRNAs, miRNAs, and mRNAs identified in this study.

**Additional file 3: Supplementary Table S3**. Summary of all DE lncRNAs, DE circRNAs, DE miRNA and DE mRNAs identified in this study.

**Additional file 4: Supplementary Table S4**. DE mRNAs of each WGCNA modules.

**Additional file 5: Supplementary Table S5**. miRNA-RNA targets predicted by Starbase.

**Additional file 6: Supplementary Table S6**. miRNA-RNA targets predicted by miRanda.

**Additional file 7: Supplementary Fig. S1**. Gross observation, Microscopic observation of liver from mice with liver fibrosis A Gross observation of liver from mice with liver fibrosis. B Hematoxylin and eosin staining (×100). C Masson staining (×100). D-F Ki-67 immunohistochemistry at 0 h (D), 12 h (E), and 72 h (F) after hepatectomy.

**Additional file 8: Supplementary Fig. S2**. Volcano plots of differentially expressed genes in the proliferative phase of liver regeneration.

**Additional file 9: Supplementary Fig. S3**. Functional analysis of lncRNA-mRNA and circRNA-mRNA core pairs. A GO enrichment analysis of lncRNA cis-regulatory targets. B KEGG enrichment analysis of lncRNA cis-regulatory targets C GO enrichment analysis of lncRNA trans-regulatory targets. D KEGG enrichment analysis of lncRNA trans-regulatory targets. E GO enrichment analysis of circRNA parental genes.

## Data Availability

The datasets supporting the conclusions of this article are included within the article and its additional files. The transcriptome sequencing data publicly available at BioProject database under the BioProject ID PRJNA953495.
