# Supplementary material for: Integrated analysis of lncRNA/circRNA–miRNA–mRNA in the proliferative phase of liver regeneration in mice with liver fibrosis
Source: BMC Genomics. 2023 Jul 24;24:417. doi: 10.1186/s12864-023-09478-z (PMC10364436; doi:10.1186/s12864-023-09478-z)
Supplement: Supplementary file 1 — Additional file 1: Supplementary Table S1. Sequences of PCR primers. [file 12864_2023_9478_MOESM1_ESM.docx]

**Supplementary Table S1. Sequences of PCR primers.**

| RNA | Primer sequence |
| --- | --- |
| Xist（NR_001463.3） | Forword TAAAGGCCTATGGCCCAGAAC  Reverse GGGGCAGGTCTCATCTTCG |
| miR-144-3p | TACAGTATAGATGATGTACT |
| Aplp2 | Forword ACTTCGACCTCTCCAAGGGA  Reverse CATCATTGGTTGGCAGGGGA |
| circ_0000117 | Forword ACCAACACTAGGATGCTGACC  Reverse ACACAGCACATTCACATGGAC |
| miR-204-5p | TTCCCTTTGTCATCCTATGCCT |
| Derl1 | Forword GGACTCGTTGCTAGGCACTT  Reverse GTCAGTTCACCCCTCGGAAG |
