# Supplementary figures and images for: Integrated analysis of lncRNA/circRNA–miRNA–mRNA in the proliferative phase of liver regeneration in mice with liver fibrosis
Source: BMC Genomics. 2023 Jul 24;24:417. doi: 10.1186/s12864-023-09478-z (PMC10364436; doi:10.1186/s12864-023-09478-z)

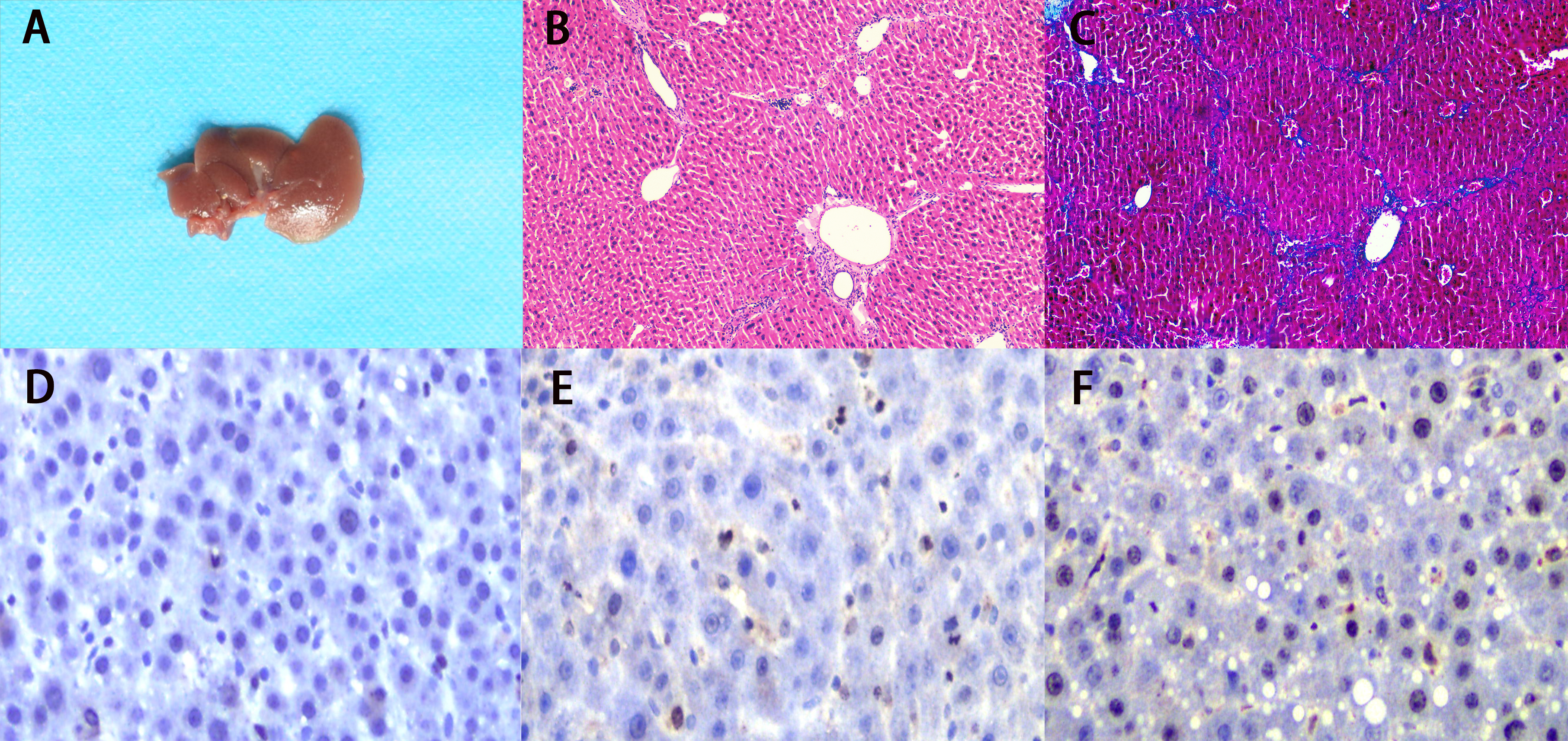

Supplement: Supplementary file 7 — Additional file 7: Supplementary Fig. S1. Gross observation, Microscopic observation of liver from mice with liver fibrosis A Gross observation of liver from mice with liver fibrosis. B Hematoxylin and eosin staining (×100). C Masson staining (×100). D-F Ki-67 immunohistochemistry at 0 h (D), 12 h (E), and 72 h (F) after hepatectomy. [file 12864_2023_9478_MOESM7_ESM.png]

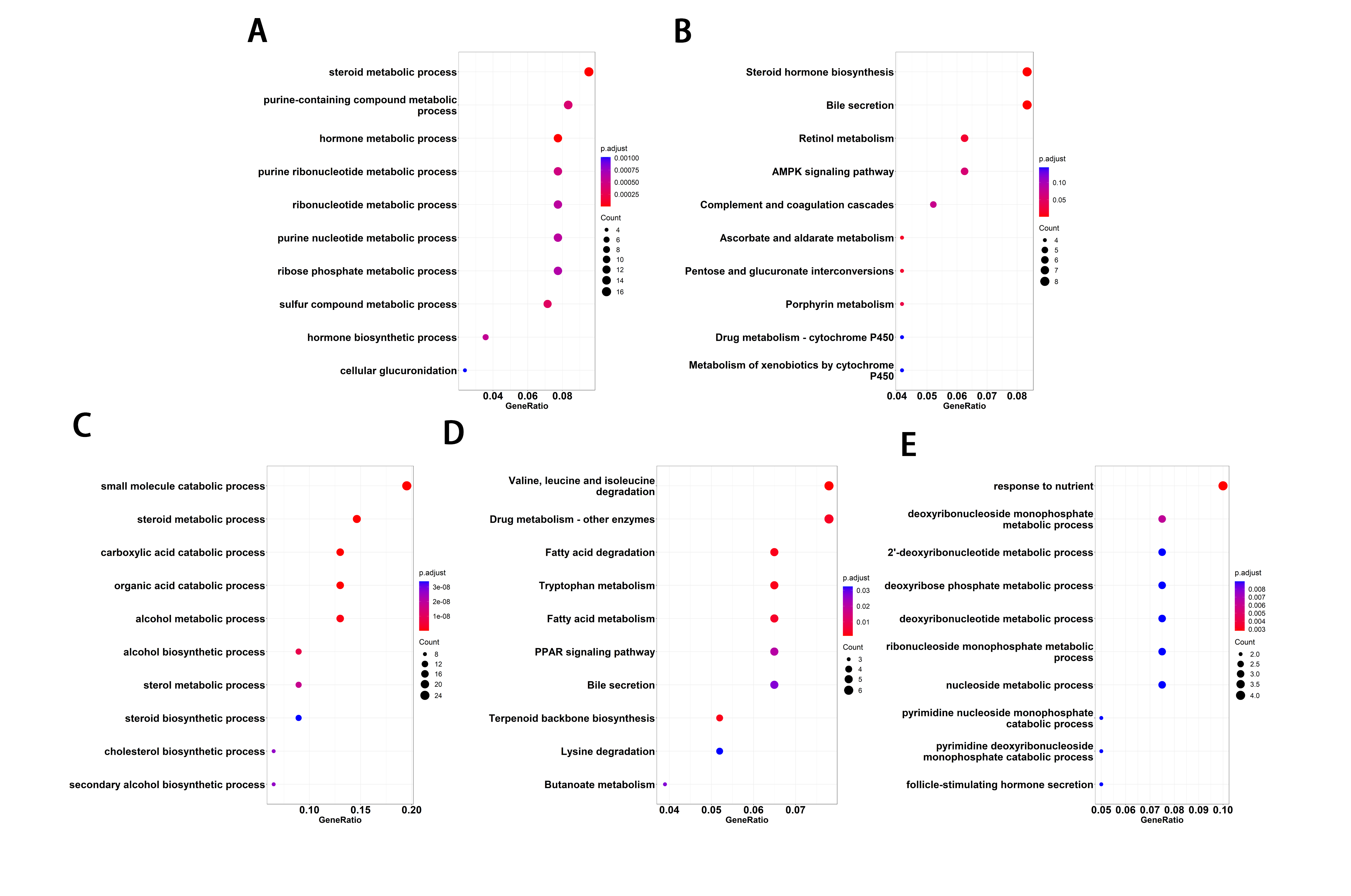

Supplement: Supplementary file 9 — Additional file 9: Supplementary Fig. S3. Functional analysis of lncRNA-mRNA and circRNA-mRNA core pairs. A GO enrichment analysis of lncRNA cis-regulatory targets. B KEGG enrichment analysis of lncRNA cis-regulatory targets C GO enrichment analysis of lncRNA trans-regulatory targets. D KEGG enrichment analysis of lncRNA trans-regulatory targets. E GO enrichment analysis of circRNA parental genes. [file 12864_2023_9478_MOESM9_ESM.png]
